# Implementing the NCTSN Trauma-Informed Organizational Assessment (TIOA) for Improving Trauma-Informed Care in Inpatient Child Psychiatry

**DOI:** 10.1177/10783903231171590

**Published:** 2023-10-18

**Authors:** Kristen Choi, Leilanie Ayala, Rebecca Lierly, Daniela Bustamante, Benjamin Cioppa-Fong, Meredith Mead, Hagop J. Mkroyan, Elizabeth Morris, Irina Babajanyan, Daniel Maryanov

**Affiliations:** 1 Kristen Choi, PhD, RN, FAAN, University of California, Los Angeles, Los Angeles, CA, USA; Gateways Hospital and Mental Health Center, Los Angeles, CA, USA; 2 Leilanie Ayala, PMHNP-BC, Sutter Center for Psychiatry, Sacramento, CA, USA; University of California, Los Angeles, Los Angeles, CA, USA; 3 Rebecca Lierly, PhD, Sutter Center for Psychiatry, Sacramento, CA, USA; 4 Daniela Bustamante, MSN, PMHNP-BC, Gateways Hospital and Mental Health Center, Los Angeles, CA, USA; 5 Benjamin Cioppa-Fong, MSN, PMHNP-BC, Gateways Hospital and Mental Health Center, Los Angeles, CA, USA; 6 Meredith Mead, MSN, PMHNP-BC, Gateways Hospital and Mental Health Center, Los Angeles, CA, USA; 7 Hagop J. Mkroyan, MSN, PMHNP-BC, Nutrix Care Partners, Los Angeles, CA, USA; 8 Elizabeth Morris, PsyD, Gateways Hospital and Mental Health Center, Los Angeles, CA, USA; 9 Irina Babajanyan, AMFT, Gateways Hospital and Mental Health Center, Los Angeles, CA, USA; 10 Daniel Maryanov, MSN, RN, Gateways Hospital and Mental Health Center, Los Angeles, CA, USA

**Keywords:** trauma-informed care, quality improvement, child and adolescent psychiatry, inpatient issues

## Abstract

**Introduction:**

Children and adolescents receiving inpatient psychiatric services have disproportionately high levels of exposure to trauma and adversity. The National Child Traumatic Stress Network Trauma-Informed Organizational Assessment (TIOA) is a comprehensive tool intended to guide implementation of trauma-informed care, but it has not yet been applied in inpatient settings.

**Aims:**

The purpose of this quality improvement project was to describe trauma-informed care in inpatient child/adolescent psychiatry with the TIOA, examine relatedness among trauma-informed care domains, and explore barriers or facilitators to applying trauma-informed care.

**Methods:**

This quality improvement project used mixed methods. We conducted a web-based survey in Summer 2022 with staff members (clinical and administrative) at two inpatient child/adolescent psychiatric units in California to assess trauma-informed care practices with the TIOA (87 items). Qualitative follow-up interviews were offered to interested participants. A correlation matrix and cluster analyses were used to examine relationships among TIOA domains; qualitative data were analyzed thematically.

**Results:**

There were 69 survey respondents and seven qualitative interviews. TIOA domain scores ranged from a low of 2.3 to a high of 3.2, indicating that practices were occurring only “rarely” to “sometimes.” There were two major themes identified from qualitive interviews: (a) barriers to trauma-informed care in an inpatient context that can be resource-constrained or coercive; and (b) discovering strategies to provide trauma-informed care despite structural barriers.

**Conclusion:**

Organizational interventions targeting any domains of trauma-informed care are needed in inpatient settings given limited uptake of trauma-informed care.

## Introduction

Adolescent mental health has been declining at alarming rates in the United States, with 20.9% of adolescents ages 12 to 17 years experiencing depression and 18.8% reporting thoughts of suicide in 2019 (Bitsko et al., 2022). The COVID-19 pandemic increased the severity of mental illness in adolescents for those already suffering and exposed a greater number of teenagers to stress (Hertz & Barrios, 2021). Adolescents who have experienced trauma and other adverse childhood experiences (ACEs) are at greater risk for developing mental illness (Bomysoad & Francis, 2020). Trauma and ACEs are associated with increased risk for suicide attempts in adolescent populations (Isohookana et al., 2013; Li et al., 2021; Miché et al., 2020). Current estimates suggest that 49.8% of children and youth in the United States have experienced at least one ACE, but in inpatient treatment settings, 95% of children and youth have experienced at least one ACE and 55% experience four or more (Havens et al., 2012; Hernandez et al., 2022). One study found that over 40% of adolescents in an inpatient facility had clinically significant symptoms of posttraumatic stress disorder (PTSD), and another found that these individuals have more suicidal ideation, higher frequencies of psychiatric hospitalizations, and longer hospitalization stays (Allwood et al., 2008; Koltek et al., 1998). Youth with PTSD in inpatient treatment settings also tend to have higher levels of clinical severity and complexity (Belivanaki et al., 2017). During hospitalization, untreated trauma symptoms and exposure to trauma reminders can trigger aggressive behaviors and violent outbursts, which might result in restrictive interventions such as seclusion and restraint application that are dangerous and re-traumatizing to the patients (Timbo et al., 2016).

Trauma-informed care is a relational health care approach that is founded on understanding of the relationship between trauma and its negative effects on short- and long-term health outcomes (Substance Abuse and Mental Health Services Administration [SAMHSA], 2014). The U.S. SAMHSA has recommended trauma-informed care for all child- and adolescent-serving organizations, in recognition of the high prevalence of trauma in clinical settings and importance of addressing trauma to promote recovery. SAMHSA’s concept of trauma-informed care includes (a) realizing how trauma affects people and their health; (b) recognizing the signs and symptoms of traumatic stress; (c) responding with trauma-informed care at all levels of an organization; and (d) resisting triggers and re-traumatization that might inadvertently occur in the process of routine care delivery (SAMHSA, 2014). A trauma-informed care approach emphasizes safety, trustworthiness and transparency, peer support, collaboration and mutuality, empowerment and choice, and awareness of the intersectional influence of culture, history, and gender (SAMHSA, 2014). This framework for service delivery can be operationalized in any setting.

Infusion of trauma-informed care principles is vital in adolescent inpatient treatment settings, given the disproportionate burden of ACEs among this population. However, there is very limited information about the extent to which inpatient treatment settings have adopted trauma-informed care as a framework for service delivery or what implementation tools have been applied in the United States. One evidence-based practice implementation of trauma-informed care in a single child and adolescent psychiatric unit in the United States found decreased rates of seclusion and physical holds (Hale & Wendler, 2023). Likewise, a longitudinal study examining outcomes of trauma-informed care implementation in five European residential youth facilities found reduced physiological stress among employees and reduced reports of physical aggression among youth (Schmid et al., 2020). Trauma-informed care implementation is linked to increased staff satisfaction, which is further linked to improved staff retention rates and improved performance (Hales et al., 2017).

Known strategies that can facilitate the adoption of trauma-informed care in inpatient and residential settings include commitment from senior leadership teams, staff support, engaging patients and families in the trauma-informed care program, aligning procedures and policies with trauma-informed care principles, and utilizing data to drive continuous change (Bryson et al., 2017). Beyond these general strategies for implementation, specific tools that can facilitate adoption of trauma-informed care in inpatient settings are limited.

Several trauma-informed care measurement tools have been developed in recent years to assess organizational policies, procedures, and culture and inform practice improvements. Some measures are designed for individual-level trauma-informed care practices, including the Attitudes Related to Trauma-Informed Care (ARTIC) Scale and Trauma-Informed Practice Scales (TIP Scales) (Baker et al., 2016; Goodman et al., 2016). Others—such as Creating Cultures of Trauma-Informed Care (CCTIC), Trauma-Informed System Change Instrument (TISCI), and the TICOMETER—are intended to guide organizational change toward implementation of trauma-informed care (Bassuk et al., 2017; Harris & Fallot, 2001; Richardson et al., 2012). Among these individual and organization measures of trauma-informed care, there is very little evidence documenting their application in inpatient psychiatric settings to guide implementation of trauma-informed care.

The National Traumatic Child Stress Network (NTCSN) has developed a new tool, the Trauma-Informed Organizational Assessment (TIOA), for organizations working with children/adolescents and families to self-assess trauma-informed care practices (Halladay Goldman et al., 2019). The TIOA improves upon existing organizational measures of trauma-informed care in several key ways. In a recent systematic review of 49 different systems-focused assessment tools, the TIOA had the highest number of assessment questions and addressed more content areas, indicating a more comprehensive assessment (Champine et al., 2019). It was designed to be used by any child/adolescent-serving organization and was developed with multi-sector trauma experts. The TIOA is a freely available tool, improving access by organizations and systems without an allotted budget for assessment measures, thereby increasing the opportunity for organizations to self-assess and utilize as part of their quality improvement processes. It includes an implementation toolkit and is specifically intended to guide trauma-informed care improvements at an organizational level (Halladay Goldman et al., 2019). Items in the TIOA reflect specific practices that trauma-informed organizations should implement, which were defined by a modified Delphi panel of approximately 90 NCTSN member trauma experts during the development of the TIOA (Halladay Goldman et al., 2019).

The TIOA is a relatively new organizational change tool to support implementation of trauma-informed care. At present, it has not been extensively used to improve trauma-informed care in inpatient child/adolescent psychiatric settings outside of the initial pilot testing of the TIOA. Applying the TIOA could facilitate adoption of trauma-informed care in a setting where this approach is most acutely needed and where highly trauma-exposed children and adolescents have the most potential to benefit. We implemented the TIOA as the first phase of a quality improvement effort in two inpatient child/adolescent psychiatry units in California, as there is little-to-no-existing evidence to guide trauma-informed care practice improvement in this setting. Our overall quality improvement project goal in using the TIOA was to assess baseline trauma-informed care and identify targets for subsequent interventions to improve trauma-informed care. Trauma-informed care interventions targeted toward one TIOA domain could result in simultaneous improvements in other related domains (e.g., workforce training on trauma-informed care may result in increased workforce knowledge as well as improvements in screening and assessment). As such, we sought to understand which domains of the TIOA were related to one another. Our baseline assessment project with the TIOA used mixed methods to describe trauma-informed care practices in inpatient psychiatry, examine relatedness among trauma-informed care domains to inform simultaneous targets for interventions, and explore barriers or facilitators to applying trauma-informed care in practice.

## Method

### Design

This was a mixed methods quality improvement project conducted in Summer 2022 with two inpatient child/adolescent psychiatric units in California. The project was determined to be exempt from IRB oversight by the UCLA IRB as quality improvement work. Prior to beginning the survey, we met with an interdisciplinary staff committee from each site to identify organizational priorities around trauma-informed care, suggestions for TIOA data collection, and long-term goals for improving the quality of trauma-informed care, consistent with recommendations from the NCTSN TIOA administration guide around planning and organizational engagement (Halladay Goldman et al., 2019). The committees were comprised of psychologists, nurses, social workers, physicians, and unit or hospital clinical leaders to ensure representation of multiple organizational and disciplinary perspectives. Our goal in meeting with these stakeholders was to assess readiness for trauma-informed care improvements, identify project champions, and solicit staff input on all phases of the project to ensure organizational buy-in. Neither of the two sites had formally implemented trauma-informed care prior to the initiation of this project, but committee members from both sites indicated strong support for the quality improvement effort.

### Sample and Setting

The setting was two inpatient child/adolescent psychiatric units in California (Los Angeles, Sacramento). The units had 24 and 23 beds, respectively, for children and adolescents 5 to 17 years of age. Each unit was part of a larger nonprofit psychiatric facility with programs for both adults and children/adolescents. The sample was clinical and administrative staff working on either unit at the time of the study, consistent with principles of trauma-informed care that all staff—not only clinical staff—should be competent in trauma-informed care (SAMHSA, 2014). Inclusion criteria were adult (>18 years of age) and current employees of either unit who had access to a web-enabled device for survey completion.

### Procedures

We administered the TIOA in a web-based survey in Summer and Fall 2022. Staff were recruited for survey participation via emails from clinical leadership and flyers with a link to the survey. Flyers contained study inclusion criteria, a brief description of the project goals, and information about the availability of survey incentives for completion of the survey and follow-up interviews. The survey took approximately 30 minutes to complete and was confidential; all participants were offered a US$25 gift card as an incentive for their time. Survey respondents had the option to indicate interest in being contacted for a brief follow-up interview.

Respondents who expressed interest in a follow-up interview were contacted to schedule an individual interview at a later date. We used a semi-structured interview guide to ask questions about trauma-informed care practices and challenges, from the practice experience of staff members, assessing barriers and facilitators to trauma-informed care. Interviews took place on Zoom with audio only and were audio-recorded for analysis. Interviews lasted 15 to 25 minutes.

### Measures

#### TIOA

The TIOA is an organizational measure of trauma-informed care developed by the NCTSN (Halladay Goldman et al., 2019). The TIOA contains a total of 87 Likert-type scale items mapping onto nine domains of trauma-informed care: screening, assessment, workforce development, resilience and protective factors, parent and caregiver trauma, cross-system collaboration, secondary traumatic stress, partnering with youth and families, and cultural responsiveness. Detailed descriptions of each TIOA domain and individual items are available elsewhere (Halladay Goldman et al., 2019). Items are scored on a 0 to 5 scale with response options for the frequency of each trauma-informed care practice: “Unable to rate,” “Never,” “Rarely,” “Sometimes,” “Most of the time,” and “Always.” Item responses within each domain were averaged to derive an overall 0 to 5 score for each of the nine TIOA domains.

#### Participant Characteristics

We asked questions about staff role and whether they had received any prior training in trauma-informed care, either in their degree programs or as continuing education (e.g., conference, webinar, and professional training). As a quality improvement project and to protect participant privacy, we did not assess detailed personal demographics.

### Analysis

Quantitative analyses were performed using R, version 4.2.1(R Core Team, 2020). Descriptive statistics were used to characterize the study sample. We used t-tests to compare differences in TIOA scores by staff role and whether or not staff had received prior trauma-informed care training. Then, we calculated Pearson *R* correlation coefficients for all TIOA overall domain scores in a correlation matrix. We considered *R* values of <.3 to indicate no correlation or a very weak correlation; .3 to .49 to indicate weak correlation; .5 to .69 to indicate moderate correlation; and greater than or equal to .7 to indicate strong correlation (Hemphill, 2003). Following development of the correlation matrix, we performed hierarchical cluster analysis on the correlation coefficients’ distance matrix with agglomerative clustering to assess relatedness among TIOA domains visually (Bridges, 2016). We considered two, three, four, five, and six cluster solutions and ultimately selected a five-cluster solution as the best conceptual fit for related domains. A dendrogram was generated to visualize hierarchical clusters and mapped onto a visual depiction of the correlation matrix in a heat map, where darker colors denoted stronger correlation. Finally, we developed a cluster plot using k-means cluster analysis on the same distance matrix to further visualize related score domains and subdomains (Malik & Tuckfield, 2019). Five conceptually meaningful clusters were apparent in both analyses (a) trauma identification (assessment and screening); (b) staff trauma-informed care factors (workforce development and secondary traumatic stress); (c) family and system factors (parent trauma, cross-system collaboration, and partnering with youth and families); (d) culture, and (e) resilience.

For analyzing qualitative data, we used Rapid Identification of Themes From Audio Recordings (RITA) procedure (Neal et al., 2015). RITA is a rapid method of qualitative data analysis that preserves critical verbal/nonverbal information and intonation of interviewees without requiring interview transcription. We selected RITA as a method for data analysis because of the quality improvement context of this project, where we intended to use findings to inform immediate next steps for practice change and not for in-depth research. RITA involves the following steps: (a) specify research foci, restricted to core aspects of the larger project for which rapid feedback is needed; (b) identify themes and create a codebook deductively from listening to audio-recordings and reviewing field notes; (c) create a coding form based on time segments or theme presence/valence; (d) test and refine the codebook and code form (e.g., revise theme definition and expand/contract time segments); and (e) code interviews (Neal et al., 2015). After developing a RITA coding framework, each interview was reviewed and coded by two independent coders, following the above process. We focused primarily on barriers or facilitators to trauma informed care.

## Results

### Sample Description

There were 69 survey respondents, seven of whom also participated in qualitative interviews. Thirty-two percent of respondents were nurses (*n* = 22), 34.8% were administrative or leadership staff (*n* = 24), and 33.3% were other clinical staff (psychologists, social workers, nurse practitioners) (*n* = 23). There were 27 respondents (39.1%) who reported prior training on trauma-informed care. The overall average TIOA score for the sample was 2.7 (*SD* = 1.5).

### TIOA Scores

**Figure 1.**
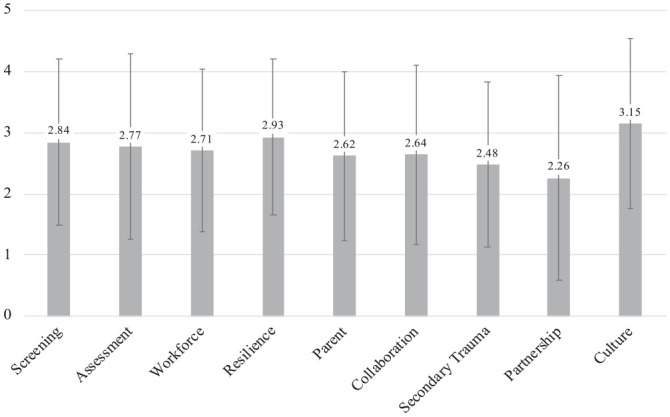
Average Trauma-Informed Organizational Assessment Domain Scores. *Note.* This figure shows the average score for each of nine domains of the National Child Traumatic Stress Network (NCTSN) Trauma-Informed Organizational Assessment (TIOA) in a sample of 69 inpatient psychiatry staff members surveyed in 2022.

Domain summary scores are shown in Figure 1. Scores ranged from a low of 2.3 (partnering with youth and families) to a high of 3.2 (cultural responsiveness), indicating that practices were occurring only “rarely” (scores of 2) to “sometimes” (scores of 3) across all domains. In bivariate tests comparing TIOA domain scores, there were no significant differences in any scores by role (clinical vs administrative staff) or prior training (yes/no).

### TIOA Domain Correlation

**Figure 2.**
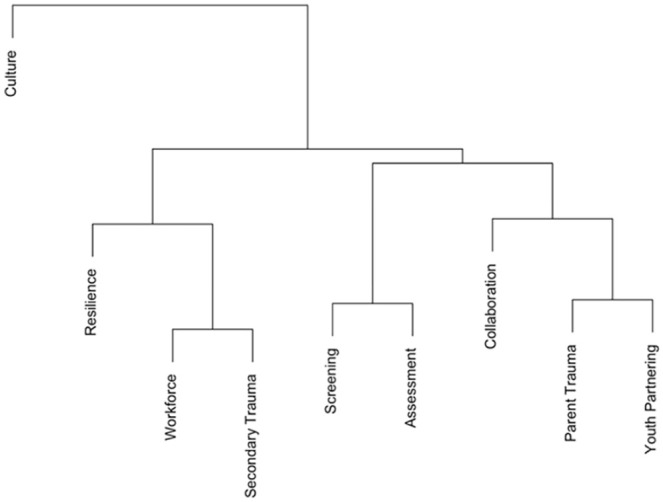
Agglomerative Hierarchical Clustering of Trauma-Informed Organizational Assessment Domain Correlations. *Note.* This dendrogram shows clustering of the nine domains of the National Child Traumatic Stress Network (NCTSN) Trauma-Informed Organizational Assessment (TIOA) in a sample of 69 inpatient psychiatry staff members surveyed in 2022.

**Figure 3.**
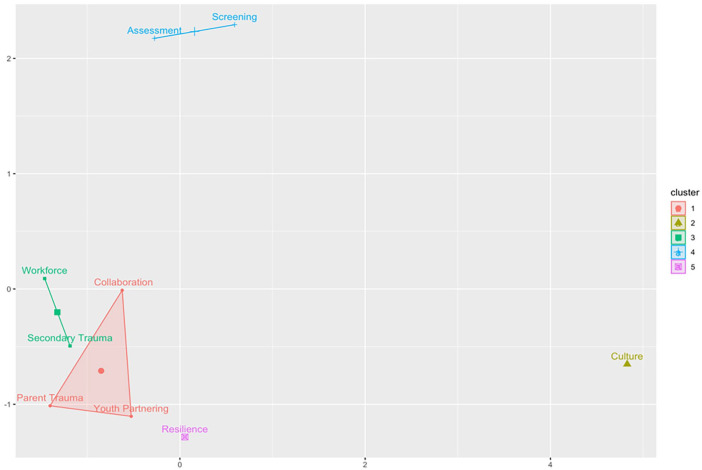
K-Means Clustering of Trauma-Informed Organizational Assessment Domain Correlations. *Note.* This figure shows a five-cluster solution to k-means clustering of correlations among the nine domains of the National Child Traumatic Stress Network (NCTSN) Trauma-Informed Organizational Assessment (TIOA). Data were derived from a sample of 69 inpatient psychiatry staff members surveyed in 2022.

The internal consistency reliability of the TIOA overall was high, with a Cronbach’s alpha value of 0.98 (95% CI = [0.96, 0.99]). In the correlation matrix, all TIOA score domains were strongly correlated with one another with Pearson *R* values greater than .7 for all correlations (Table 1). All correlations were statistically significant. In hierarchical and *k*-means clustering (Figures 2 and 3), there was evidence for clustering of correlations among the following domains: assessment and screening; workforce development and secondary traumatic stress; and parent trauma, cross-system collaboration, and partnering with youth and families. Strengthening resilience and protective factors and cultural responsiveness domains were single-domain clusters that did not cluster strongly with any of the other three clusters.

**Table 1.** Trauma-Informed Organizational Assessment Domain Score Correlation Matrix.

Correlation coefficients	Assessment	Workforce development	Resilience and protective factors	Parent and caregiver trauma	Cross-system collaboration	Secondary traumatic stress	Partnering with youth and families	Cultural responsiveness
Screening	.92*	.88*	.84*	.86*	.83*	.82*	.83*	.79*
Assessment		.88*	.82*	.86*	.86*	.89*	.85*	.78*
Workforce development			.90*	.88*	.90*	.94*	.86*	.81*
Resilience and protective factors				.89*	.80*	.87*	.84*	.79*
Parent and caregiver trauma					.89*	.89*	.92*	.78*
Cross-system collaboration						.86*	.85*	.76*
Secondary traumatic stress							.86*	.79*
Partnering with youth and families								.77*

*Note.* Pearson *R* correlation coefficients.

* *p* < .01.

### Qualitative Themes

There were two major themes identified from qualitative interviews: (a) barriers to trauma-informed care in an inpatient context that can be resource-constrained or coercive; and (b) discovering strategies to provide trauma-informed care despite structural barriers. The barriers and facilitators described by participants are described in detail and summarized in Table 2.

**Table 2.** Barriers and Facilitators to Trauma-Informed Care.

Barriers	Facilitators
• Lack of formal trauma-informed care training • Need to prioritize crisis response in an inpatient setting • No formal trauma assessment process • Organizational resource constraints • Short patient stays and insufficient capacity for long-term follow up • Invasive safety monitoring • Staffing challenges • Presence and use of seclusion/restraints • Harsh physical environment	• Staff awareness of common trauma symptoms and triggers • Establishing therapeutic relationships • Building trust with patients • Communicating understanding for patients’ points of view • Setting limits and boundaries that promote a sense of safety • Teamwork and team communication • Empathetic language and nonjudgement • Awareness of nonverbal communication

### Theme: Barriers to Trauma-Informed Care in an Inpatient Context That Can Be Resource-Strained or Coercive

Respondents identified a lack of formal trauma-informed care training, the absence of formal processes for assessment or intervention, and always being in “crisis stabilization” mode as key barriers to trauma-informed care. Staff members felt that trauma exposures and triggers were often “missed” due to a lack of formal assessment or intervention processes. One staff member saw trauma-informed practice as “up to the luck of the draw whether a staff member has been exposed to the concept of trauma informed care,” noting a lack of training options for staff. In addition, a nurse described their unit as “reactive” due to resource constraints (e.g., inadequate staff or programming), with insufficient resources to go beyond basic safety and crisis management. Another staff member noted,

We want to help the patients manage their symptoms of trauma without cracking open a huge can of worms . . . if they’re only here for crisis stabilization, maybe for five to seven days, that’s certainly not long enough to process trauma.

Multiple participants were unsure of “how to do more than a bandaid,” in the words of one interviewee, in a short-term, crisis-management context.

The difficulty of avoiding trauma triggers in an inpatient setting was discussed in several interviews. Participants noted the required “constant” monitoring of patients for safety checks, typically every 15 minutes, that did not allow patients a great deal of privacy. They also discussed staffing challenges, such as when male staff members were assigned to monitor patients with prior trauma exposures from men but there were insufficient staff to make reassignments. A participant said, “There is a lot of fear being around staff members, sometimes of specific genders or of different racial groups.” Another trauma trigger identified by participants was seclusion and restraints, described by one participant as “incredibly triggering for people who have been through trauma, who have been held down, who have been through abuse that involved having hands placed on them in some way or being restrained in some way that was not wanted.” Likewise, a participant noted of restraint use,

I don’t always feel like de-escalation is handled the best way and it really varies based on the practitioner. . . it definitely does not establish a sense of trust when you know you’re going to just get sedated.

Finally, participants noted physical aspects of the unit, such as poor lighting, the absence of comforting items, and frequent loud noises and echoing hard surfaces that could be triggering. They described inpatient units as “stark,” “harsh,” “institutional,” “creepy,” and “bare,” lacking a sense of warmth, comfort, and physical safety that would be beneficial to youth with a trauma history.

### Theme: Discovering Strategies to Provide Trauma-Informed Inpatient Care Despite Structural Barriers

Despite a lack of formal trauma-informed care training among interview participants, they recounted strategies they have developed informally that align with principles of trauma-informed care assessed in the TIOA. Participants were sensitized to the signs and symptoms of trauma and aware of how to identify its many possible presentations among children and adolescents. They were aware that trauma could look like crying, withdrawal, dysphoria, guardedness, anxiety, and fear; but could also manifest as reactivity, verbal and physical aggression, or other acting out behaviors.

Participants emphasized the importance of establishing “a therapeutic relationship as a non-threat and somebody who’s not necessarily going to lecture, or be stern or punish.” Participants believed that “gaining trust” and helping patients to “drop their guard” was essential to forming a therapeutic relationship. One participant said,

Trying to get their point of view is a big way of gaining their trust I think, regularly checking in and then just making sure that you’re listening and responding to their needs.

While participants recognized the importance of gaining trust, they also noted a need to “be the adult” in the room. A participant described this delicate relational balance as, “It’s being relatable to a young person in a kind, friendly way, but also not in a way because they want you to be the adult. They want the safety.” Another participant recalled a coworker who was especially skilled at balancing the boundaries that are needed for safety with building a trusting relationship, saying of their coworker,

I’m thinking of this one [coworker who’s] gone through lots of trauma [themself]. I see [their] ability to listen and be firm and also coax things out of kids that we as nurses may have not been able to.”

Teamwork and communication were viewed as essential to appropriate care of patients with a trauma history. A participant said of communication in the context of trauma-informed care: “It’s a must.” They described treatment planning and centralizing communication with the charge nurse as effective strategies for ensuring that all staff members were aware of new trauma disclosures or trauma-related needs. Other interpersonal practice skills that staff found to be effective for working with youth exposed to trauma were using empathetic language, communicating openly and honestly about care, nonjudgement, listening, awareness of nonverbal communication such as body language, attentiveness, and interacting with youth at the same physical level (e.g., not standing above them).

## Discussion

This project assessed trauma-informed organizational practices in inpatient child/adolescent psychiatry to define targets for quality improvement. We found that trauma-informed care practices across nine domains of the TIOA occurred infrequently and inconsistently, with no differences in ratings by staff who did versus did not have prior training in trauma-informed care. In addition, we found that certain TIOA domains clustered together in ratings. These were conceptually related, with distinct clusters for trauma identification, staff trauma-informed care factors, and family and system factors. Cultural responsiveness and resilience/protective factors were single-domain clusters, as these two concepts were different from others and one another in our analysis. The clusters suggest that certain trauma-informed care practices may lend themselves to simultaneous implementation or improvement efforts. The final two domains, strengthening resilience/protective factors and cultural responsiveness, were single-domain clusters. Partnering with youth and families in meaningful ways is a pervasive challenge in mental health research and practice, and our findings suggest that this challenge persists as this domain received the lowest scores (Faithfull et al., 2018). Our findings indicate a need for organizational interventions to improve the provision of trauma-informed care in inpatient settings, in youth partnerships and other domains.

In qualitative interviews, staff members identified barriers and challenges to trauma informed care in their inpatient child/adolescent psychiatry practice setting. Many of these barriers were structural, such as policies requiring frequent safety monitoring; a harsh physical environment (e.g., locked doors, fluorescent lighting); insufficient staff training; the absence of clear processes for trauma assessment and intervention; and the availability of seclusion or restraints (Chandler, 2008). One participant discussed not being able to assure racial or gender concordance between patients and staff members as a barrier to trauma-informed care, though it should be noted that not all requests for a provider of a different race or gender stem from trauma but may instead be manifestations of prejudice or discrimination (Paul-Emile et al., 2016). Guides for distinguishing the appropriateness of patient requests for a provider of a different race or gender are available to navigate such requests (Paul-Emile et al., 2016). Even without formal training, staff members also described interpersonal practices they used to build trust and a sense of safety with patients within these contextual constraints. Although past research on this topic is limited, our qualitative findings align with other studies on the challenges of trauma-informed care in structured settings, particularly the structural and organizational barriers that must be overcome to delivery trauma-informed care (O’Dwyer et al., 2021; Wilson et al., 2017). Together, our quantitative and qualitative findings suggest a need for organizational interventions to improve the delivery of trauma-informed care (Bryson et al., 2017).

When implementing trauma-informed care, best practices include securing commitment from senior leadership teams, engaging front-line staff, engaging patients and families, aligning procedures and policies with trauma-informed care principles, and utilizing data to drive continuous change (Bryson et al., 2017). Although staff training to increase awareness of traumatic stress and effective interpersonal practices for trauma response are warranted, organizational interventions that go beyond individual staff training and those that align with the above best practices for stakeholder engagement and organizational restructuring will be required for sustained practice improvement (Bryson et al., 2017). Such changes could include embedding trauma screening or assessments in electronic medical records; providing occupational health and leave resources for secondary traumatic stress; offering regular staff trainings on trauma-informed care, including embedding training in onboarding for new staff; modifying the physical environment to create spaces with a sense of safety; investing in alternatives to seclusion or restraint; and establishing collaborative relationships with multi-sector partner organizations. Trauma-informed care practice improvements should involve not only front-line staff, but input from patients and families to ensure that services are responsive to youth-identified needs (Faithfull et al., 2018). Finally, it is important for leadership and administrative staff to also receive training in trauma-informed care to ensure that they can support staff in operationalizing this approach with patients.

In light of findings from the initial phase of this quality improvement project to address trauma in inpatient child/adolescent psychiatry, we aim to systematically address each domain of the TIOA with both clinician-level and organization-level interventions, beginning with the screening and assessment domains. Project next steps include embedding trauma screening and assessment into the electronic health record, establishing care processes for identification of trauma, and implementing ongoing staff training on trauma-informed care.

There are strengths and limitations to this project. We assessed trauma-informed care with a validated, comprehensive organizational measure that was designed for practice improvement. Our project involved two inpatient sites in Northern and Southern California, and we had adequate representation of multiple clinical disciplines among survey respondents. Limitations of the study were the absence of a control group or benchmark against which to compare TIOA scores; a voluntary response sampling frame; and the possibility of reporting bias in both the survey and interviews. The sample size was relatively small, limiting power to detect small differences in domain scores. We did not directly engage patients or families at this initial stage of the project or seek patient/family perspectives on trauma-informed care, although this is a planned next step for our project. We did not assess detailed staff employment backgrounds or whether those with trauma-informed care training had worked in trauma-informed organizations. As a quality improvement project, our findings reflect our two improvement sites and may not be directly generalizable to all inpatient child/adolescent psychiatry units or all child-serving systems. Future work in this area should explore how to engage youth/families in trauma-informed care improvements and evaluate organizational interventions for their influence on TIOA scores as well as youth perceptions of trauma-informed care.

## Conclusion

Trauma-informed care is essential in inpatient child/adolescent psychiatric settings, given the disproportionately high prevalence of trauma in clinical populations. However, our quality improvement project using the TIOA found trauma-informed care is lacking. Findings from this project may be used to define targets for future quality improvement efforts to build a stronger culture of trauma-informed care in inpatient child/adolescent psychiatry. Based on domain scores identified in our baseline assessment, we plan to target workforce development/secondary traumatic stress, partnering with youth and families, and developing formal screening/assessment procedures. As the evidence base for organizational interventions for trauma-informed care in this setting grows, the TIOA implementation toolkit and other repositories for trauma-informed care resources should grow to ensure that interventions are disseminated nationally.

## References

[bibr1-10783903231171590] Allwood M. A. Dyl J. Hunt J. I. Spirito A. (2008). Comorbidity and service utilization among psychiatrically hospitalized adolescents with posttraumatic stress disorder. Journal of Psychological Trauma, 7(2), 104–121. 10.1080/19322880802231791

[bibr2-10783903231171590] Baker C. N. Brown S. M. Wilcox P. D. Overstreet S. Arora P. (2016). Development and psychometric evaluation of the Attitudes Related to Trauma-Informed Care (ARTIC) Scale. School Mental Health, 8(1), 61–76. 10.1007/S12310-015-9161-0/TABLES/4

[bibr3-10783903231171590] Bassuk E. L. Unick G. J. Paquette K. Richard M. K. (2017). Developing an instrument to measure organizational trauma-informed care in human services: The TICOMETER. Psychology of Violence, 7(1), 150–157. https://psycnet.apa.org/buy/2016-03236-001

[bibr4-10783903231171590] Belivanaki M. Ropi S. Kanari N. Tsiantis J. Kolaitis G. (2017). Trauma and post-traumatic stress disorder among psychiatric inpatient children and adolescents. European Journal of Psychotraumatology, 8(Suppl. 4), 1351161. 10.1080/20008198.2017.1351161

[bibr5-10783903231171590] Bitsko R. H. Claussen A. H. Lichstein J. Black L. I. Jones S. E. Danielson M. L. Hoenig J. M. Davis Jack S. P. Brody D. J. Gyawali S. Maenner M. J. Warner M. Holland K. M. Perou R. Crosby A. E. Blumberg S. J. Avenevoli S. Kaminski J. W. Ghandour R. M. (2022). Mental health surveillance among children—United States, 2013–2019. MMWR Supplements, 71(2), 1–42. 10.15585/MMWR.SU7102A1 PMC8890771 35202359

[bibr6-10783903231171590] Bomysoad R. N. Francis L. A. (2020). Adverse childhood experiences and mental health conditions among adolescents. Journal of Adolescent Health, 67(6), 868–870. 10.1016/J.JADOHEALTH.2020.04.013 32576484

[bibr7-10783903231171590] Bridges C. C. (2016). Hierarchical cluster analysis. Psychological Reports, 18(3), 851–854. 10.2466/PR0.1966.18.3.851

[bibr8-10783903231171590] Bryson S. A. Gauvin E. Jamieson A. Rathgeber M. Faulkner-Gibson L. Bell S. Davidson J. Russel J. Burke S. (2017). What are effective strategies for implementing trauma-informed care in youth inpatient psychiatric and residential treatment settings? A realist systematic review. International Journal of Mental Health Systems, 11(1), 1–16. 10.1186/S13033-017-0137-3 28503194 PMC5425975

[bibr9-10783903231171590] Champine R. B. Lang J. M. Nelson A. M. Hanson R. F. Tebes J. K. (2019). Systems measures of a trauma-informed approach: A systematic review. American Journal of Community Psychology, 64(3–4), 418–437. 10.1002/AJCP.12388 31469452 PMC7003149

[bibr10-10783903231171590] Chandler G. (2008). From traditional inpatient to trauma-informed treatment: Transferring control from staff to patient. Journal of the American Psychiatric Nurses Association, 14(5), 363–371. 10.1177/1078390308326625 21665779

[bibr11-10783903231171590] Faithfull S. Brophy L. Pennell K. Simmons M. B. (2018). Barriers and enablers to meaningful youth participation in mental health research: Qualitative interviews with youth mental health researchers. Journal of Mental Health, 28(1), 56–63. 10.1080/09638237.2018.1521926 30353772

[bibr12-10783903231171590] Goodman L. A. Sullivan C. M. Serrata J. Perilla J. Wilson J. M. Fauci J. E. DiGiovanni C. D. (2016). Development and validation of the trauma-informed practice scales. Journal of Community Psychology, 44(6), 747–764. 10.1002/JCOP.21799

[bibr13-10783903231171590] Hale R. Wendler M. C. (2023). Evidence-based practice: Implementing trauma-informed care of children and adolescents in the inpatient psychiatric setting. Journal of the American Psychiatric Nurses Association, 29, 161–170. 10.1177/1078390320980045 33349098

[bibr14-10783903231171590] Hales T. W. Nochajski T. H. Green S. A. Hitzel H. K. Woike-Ganga E. (2017). An association between implementing trauma-informed care and staff satisfaction. Advances in Social Work, 18(1), 300–312. 10.18060/21299

[bibr15-10783903231171590] Halladay Goldman J. Purbeck Trunzo C. Agosti J . (2019). Trauma-informed organizational assessment (TIOA) information packet. National Center for Child Traumatic Stress. https://www.nctsn.org/resources/trauma-informed-organizational-assessment-information-packet 10.1177/10775595251399911 41275411

[bibr16-10783903231171590] Harris M. Fallot R. (2001). Creating Cultures of Trauma-Informed Care (CCTIC): A self-assessment and planning protocol. Jossey-Bass. https://calio.dspacedirect.org/handle/11212/4468

[bibr17-10783903231171590] Havens J. F. Gudiño O. G. Biggs E. A. Diamond U. N. Weis J. R. Cloitre M. (2012). Identification of trauma exposure and PTSD in adolescent psychiatric inpatients: An exploratory study. Journal of Traumatic Stress, 25(2), 171–178. 10.1002/JTS.21683 22522731 PMC3742006

[bibr18-10783903231171590] Hemphill J. F. (2003). Interpreting the magnitudes of correlation coefficients. American Psychologist, 58(1), 78–79. 10.1037/0003-066X.58.1.78 12674822

[bibr19-10783903231171590] Hernandez K. William T. B. College J. Andoni L. Hellmuth J. Holler K. Sullivan S. (2022). Adverse childhood experiences (ACEs), depression, and suicidality in young children admitted to a psychiatric hospital. 10.21203/rs.3.rs-1659403/v1

[bibr20-10783903231171590] Hertz M. F. Barrios L. C. (2021). Adolescent mental health, COVID-19, and the value of school-community partnerships. Injury Prevention, 27(1), 85–86. 10.1136/INJURYPREV-2020-044050 33172840

[bibr21-10783903231171590] Isohookana R. Riala K. Hakko H. Räsänen P. (2013). Adverse childhood experiences and suicidal behavior of adolescent psychiatric inpatients. European Child and Adolescent Psychiatry, 22(1), 13–22. 10.1007/S00787-012-0311-8/TABLES/4 22842795

[bibr22-10783903231171590] Koltek M. Wilkes T. C. R. Atkinson M. (1998). The prevalence of posttraumatic stress disorder in an adolescent inpatient unit. The Canadian Journal of Psychiatry, 43(1), 64–68. 10.1177/070674379804300107 9494749

[bibr23-10783903231171590] Li S. Wang S. Gao X. Jiang Z. Xu H. Zhang S. Sun Y. Tao F. Chen R. Wan Y. (2021). Patterns of adverse childhood experiences and suicidal behaviors in adolescents: A four-province study in China. Journal of Affective Disorders, 285, 69–76. 10.1016/J.JAD.2021.02.045 33636673

[bibr24-10783903231171590] Malik A. Tuckfield B. (2019). Applied unsupervised learning with R. Packt Publishing.

[bibr25-10783903231171590] Miché M. Hofer P. D. Voss C. Meyer A. H. Gloster A. T. Beesdo-Baum K. Wittchen H. U. Lieb R. (2020). Specific traumatic events elevate the risk of a suicide attempt in a 10-year longitudinal community study on adolescents and young adults. European Child and Adolescent Psychiatry, 29(2), 179–186. 10.1007/S00787-019-01335-3/TABLES/2 31054127

[bibr26-10783903231171590] Neal J. W. Neal Z. P. VanDyke E. Kornbluh M. (2015). Expediting the analysis of qualitative data in evaluation: A procedure for the Rapid Identification of Themes From Audio Recordings (RITA). American Journal of Evaluation, 36(1), 118–132. 10.1177/1098214014536601/ASSET/IMAGES/LARGE/10.1177_1098214014536601-FIG2.JPEG

[bibr27-10783903231171590] O’Dwyer C. Tarzia L. Fernbacher S. Hegarty K. (2021). Health professionals’ experiences of providing trauma-informed care in acute psychiatric inpatient settings: A scoping review. Trauma, Violence, & Abuse, 22(5), 1057–1067. 10.1177/1524838020903064 32027227

[bibr28-10783903231171590] Paul-Emile K. Smith A. K. Lo B. Fernández A. (2016). Dealing with racist patients. New England Journal of Medicine, 374, 708–711. 26933847 10.1056/NEJMp1514939

[bibr29-10783903231171590] R Core Team. (2020). R: A language and environment for statistical computing. R Foundation for Statistical Computing.

[bibr30-10783903231171590] Richardson M. M. Coryn C. L. S. Henry J. Black-Pond C. Unrau Y. (2012). Development and evaluation of the trauma-informed system change instrument: Factorial validity and implications for use. Child and Adolescent Social Work Journal, 29(3), 167–184. 10.1007/S10560-012-0259-Z/TABLES/3

[bibr31-10783903231171590] Schmid M. Lüdtke J. Dolitzsch C. Fischer S. Eckert A. Fegert J. M. (2020). Effect of trauma-informed care on hair cortisol concentration in youth welfare staff and client physical aggression towards staff: Results of a longitudinal study. BMC Public Health, 20(1), 1–11. 10.1186/S12889-019-8077-2/TABLES/4 31910832 PMC6947862

[bibr32-10783903231171590] Substance Abuse and Mental Health Services Administration. (2014). SAMHSA’s concept of trauma and guidance for a trauma-informed approach. HHS Publication No. (SMA) 14-4884. https://ncsacw.acf.hhs.gov/userfiles/files/SAMHSA_Trauma.pdf

[bibr33-10783903231171590] Timbo W. Sriram A. Reynolds E. K. DeBoard-Lucas R. Specht M. Howell C. McSweeney C. Grados M. A. (2016). Risk factors for seclusion and restraint in a pediatric psychiatry day hospital. Child Psychiatry and Human Development, 47(5), 771–779. 10.1007/S10578-015-0608-1/TABLES/4 26643416

[bibr34-10783903231171590] Wilson A. Hutchinson M. Hurley J. (2017). Literature review of trauma-informed care: Implications for mental health nurses working in acute inpatient settings in Australia. International Journal of Mental Health Nursing, 26(4), 326–343. 10.1111/INM.12344 28480568

